# Ovarian cancer and smoking: individual participant meta-analysis including 28 114 women with ovarian cancer from 51 epidemiological studies

**DOI:** 10.1016/S1470-2045(12)70322-4

**Published:** 2012-09

**Authors:** Collaborative Group on Epidemiological Studies of Ovarian Cancer

## Abstract

**Background:**

Smoking has been linked to mucinous ovarian cancer, but its effects on other ovarian cancer subtypes and on overall ovarian cancer risk are unclear, and the findings from most studies with relevant data are unpublished. To assess these associations, we review the published and unpublished evidence.

**Methods:**

Eligible epidemiological studies were identified by electronic searches, review articles, and discussions with colleagues. Individual participant data for 28 114 women with and 94 942 without ovarian cancer from 51 epidemiological studies were analysed centrally, yielding adjusted relative risks (RRs) of ovarian cancer in smokers compared with never smokers.

**Findings:**

After exclusion of studies with hospital controls, in which smoking could have affected recruitment, overall ovarian cancer incidence was only slightly increased in current smokers compared with women who had never smoked (RR 1·06, 95% CI 1·01–1·11, p=0·01). Of 17 641 epithelial cancers with specified histology, 2314 (13%) were mucinous, 2360 (13%) endometrioid, 969 (5%) clear-cell, and 9086 (52%) serous. Smoking-related risks varied substantially across these subtypes (p_heterogeneity_<0·0001). For mucinous cancers, incidence was increased in current versus never smokers (1·79, 95% CI 1·60–2·00, p<0·0001), but the increase was mainly in borderline malignant rather than in fully malignant tumours (2·25, 95% CI 1·91–2·65 *vs* 1·49, 1·28–1·73; p_heterogeneity_=0·01; almost half the mucinous tumours were only borderline malignant). Both endometrioid (0·81, 95% CI 0·72–0·92, p=0·001) and clear-cell ovarian cancer risks (0·80, 95% CI 0·65–0·97, p=0·03) were reduced in current smokers, and there was no significant association for serous ovarian cancers (0·99, 95% CI 0·93–1·06, p=0·8). These associations did not vary significantly by 13 sociodemographic and personal characteristics of women including their body-mass index, parity, and use of alcohol, oral contraceptives, and menopausal hormone therapy.

**Interpretation:**

The excess of mucinous ovarian cancers in smokers, which is mainly of tumours of borderline malignancy, is roughly counterbalanced by the deficit of endometrioid and clear-cell ovarian cancers. The substantial variation in smoking-related risks by tumour subtype is important for understanding ovarian carcinogenesis.

**Funding:**

Cancer Research UK and MRC.

## Introduction

Until recently, smoking was not thought to be a risk factor for ovarian cancer, but in 2009 the International Agency for Research on Cancer added mucinous ovarian tumours (which comprise about a tenth of all ovarian cancers) to their list of tobacco-related cancers.^1^ We identified 56 epidemiological studies of ovarian cancer that obtained information about women's smoking history. Some results have been published from 55 of the 56 studies,^2^, ^3^, ^4^, ^5^, ^6^, ^7^, ^8^, ^9^, ^10^, ^11^, ^12^, ^13^, ^14^, ^15^, ^16^, ^17^, ^18^, ^19^, ^20^, ^21^, ^22^, ^23^, ^24^, ^25^, ^26^, ^27^, ^28^, ^29^, ^30^, ^31^, ^32^, ^33^, ^34^, ^35^, ^36^, ^37^, ^38^, ^39^, ^40^, ^41^, ^42^, ^43^, ^44^, ^45^, ^46^, ^47^, ^48^, ^49^, ^50^, ^51^, ^52^, ^53^, ^54^, ^55^, ^56^ but results on smoking-related risks have been published from only about a third of these studies.^4^, ^5^, ^10^, ^15^, ^16^, ^18^, ^20^, ^23^, ^27^, ^32^, ^34^, ^35^, ^43^, ^44^, ^46^, ^50^, ^53^, ^54^, ^56^ Almost all reported little or no association between smoking and overall risk of ovarian cancer; some, but not all, reported an increased risk of mucinous tumours in smokers, but not for other subtypes of ovarian cancer.

The Collaborative Group on Epidemiological Studies of Ovarian Cancer was set up to bring together and reanalyse the available epidemiological evidence, published and unpublished, on the association between various factors and ovarian cancer risk.^57^ To avoid selective emphasis on results from the few studies that have published their findings, this report sought data from all studies larger than a specific size that have obtained relevant information about the relation between ovarian cancer risk and women's smoking history, whether published or not.

## Methods

### Search strategy and selection criteria

This collaboration began in 1998, and since then potentially eligible epidemiological studies have been sought regularly by searches of review articles and from computer-aided literature searches in Medline, Embase, and PubMed, with combinations of the search terms “ovarian cancer”, “ovary cancer”, “smok*”, and “tobacco”. To be eligible for these analyses, studies needed to have obtained individual data for women's reproductive history, use of hormonal therapies, and smoking history and to have studied at least 200 women with ovarian cancer (before 2006, studies with less than 200 cases of ovarian cancer had been eligible, so there are fewer cases in some early studies). Studies that had obtained relevant data, but had not published on ovarian cancer and smoking, were sought by correspondence with colleagues, by discussions at collaborators meetings, and by electronic searches with additional terms “cohort”, “prospective”, “women”, and “cancer risk”.

We identified 56 eligible studies and invited principal investigators from each to participate in the collaboration. Investigators from two eligible studies^52^, ^53^ did not respond to our enquiries and those from three other eligible studies^54^, ^55^, ^56^ were unable to participate. Thus, data from 51 of the 56 eligible studies identified are analysed in this report, and implications of the slight incompleteness are discussed later.

### Data extraction

Cases were women with malignant epithelial (borderline malignant or fully malignant) or with non-epithelial ovarian cancer and controls were women without ovarian cancer who had not undergone bilateral oophorectomy. Information sought from principal investigators about each individual case and control included their age, ethnic group, education, alcohol and tobacco use, height, weight or body-mass index (BMI), age at menarche, reproductive history, use of hormonal contraceptives, use of menopausal hormonal therapy, hysterectomy, and family history of ovarian or breast cancer. The information sought about these factors related to the time preceding the onset of symptoms for cases and to an equivalent time for controls. So that similar analytical methods could be used across studies, we incorporated cohort studies using a nested case–control design, in which up to four controls were selected at random, matched by age at cancer diagnosis and, where appropriate, by broad geographical region. In one cohort study,^21^ cases were women with fatal ovarian cancer, whereas in all other studies cases were women with incident disease.

Principal investigators of the 51 epidemiological studies (one of which is unpublished) in these analyses^2^, ^3^, ^4^, ^5^, ^6^, ^7^, ^8^, ^9^, ^10^, ^11^, ^12^, ^13^, ^14^, ^15^, ^16^, ^17^, ^18^, ^19^, ^20^, ^21^, ^22^, ^23^, ^24^, ^25^, ^26^, ^27^, ^28^, ^29^, ^30^, ^31^, ^32^, ^33^, ^34^, ^35^, ^36^, ^37^, ^38^, ^39^, ^40^, ^41^, ^42^, ^43^, ^44^, ^45^, ^46^, ^47^, ^48^, ^49^, ^50^, ^51^ provided individual information about smoking history for cases and controls. The analyses used information provided by principal investigators about a woman's smoking history before the onset of symptoms. For retrospective case–control studies, this was smoking history before the onset of symptoms for cases and at an equivalent time for controls. For prospective studies, principal investigators generally provided information about women's smoking history recorded at the time they were recruited into the cohort. The information provided was used to classify all women as ever or never smokers, and in all but three studies^7^, ^14^, ^45^ ever smokers could be classified as either current or past smokers. Information about amount smoked and timing of exposure was sought only for the few studies that joined the collaboration after 2009, so reliable analyses of these aspects of smoking could not be done. All data contributed by principal investigators were checked and collated centrally so that analyses could use definitions that were as similar as possible across studies. Apparent inconsistencies in the data were rectified, where possible, by correspondence with the investigators. After the records had been checked and corrected, investigators were sent summary tables and listings of the variables to be used in analyses for final confirmation that their data had been correctly interpreted.

Information about histological classification and malignant potential of the ovarian cancers was sought from principal investigators. Tumours were classified as epithelial, non-epithelial, or malignant not otherwise specified (NOS). The epithelial cancers were then classified as clear-cell, endometrioid, mucinous, serous, other, mixed, or NOS, and were further subdivided by their malignant potential as borderline malignant, fully malignant, and not known whether borderline or fully malignant. If investigators provided International Classification of Diseases for Oncology^58^ codes, these were used to classify tumours centrally. Information about ovarian cancer histology was provided by investigators of all but five^9^, ^10^, ^12^, ^21^, ^36^ of the 51 participating studies; not all studies had included non-epithelial and borderline malignant ovarian cancer.

### Statistical analysis

We used conditional logistic regression to calculate the relative risk (RR) of ovarian cancer in relation to smoking history (ie, the incidence rate ratio among otherwise similar women of the same age, calculated as the ratio of the odds of smoking among cases to the odds of smoking among controls). To ensure that women in one study were compared directly only with otherwise similar women in the same study, all analyses were routinely stratified by study, by centre within study, by fine divisions of age (5-year age groups up to 85–89 years), ever use of menopausal hormonal therapy (yes, no), menopausal status or hysterectomy (premenopausal or perimenopausal, natural menopause before age 50 years, natural menopause at or after age 50 years, previous hysterectomy, other or unknown), and BMI (<25 kg/m^2^, ≥25 kg/m^2^) and were routinely adjusted by parity (0, 1–2, ≥3) and use of oral contraceptives (no or yes for durations of <5 years and ≥5 years). For other potential confounding factors (year of birth, ethnic origin, education, family history of ovarian or breast cancer, age at menarche, and alcohol use), we did sensitivity analyses comparing results before and after adjustment for each variable in turn and for all simultaneously. Unknowns for each stratification and adjustment variable were assigned to separate groups. We made comparisons across different subgroups of women using standard χ^2^ tests for heterogeneity, calculated from the change in log likelihood on adding extra terms. Significance tests for heterogeneity of the effect of smoking by tumour subtype were based on analyses within cases only, because controls provide no additional information. Smoking status was treated as a dichotomous outcome (current *vs* never) and the term for tumour subtype was treated as the variable of interest in a conditional logistic regression, stratified and adjusted as described previously.

Analyses were done using STATA (version 11). Results in the figures are presented with squares and lines. The position of the square shows the value of the RR and its area is inversely proportional to the variance of the logarithm of the RR, thereby providing an indication of the amount of statistical information available for that particular estimate. When results from many studies, many tumour subtypes, or many subgroups are presented in the figures, the lines show 99% CIs (rather than 95% CIs) to help to allow for multiple testing. When the main results are given in the text, however, 95% CIs are used.

### Role of the funding source

The funders had no role in study design, data collection, analysis or interpretation of data, preparation of the report, or the decision to publish. All members of the analysis and writing committee (VB, KG, CH, KM, RP, GR) had access to the raw data and are responsible for the final submission for publication.

## Results

Table 1 shows details of the women in the 51 participating studies. The studies are grouped by their design and, within each type of design, are ordered by the median year when the ovarian cancers were diagnosed. All but eight of the studies were done in North America or Europe, and all but six in high-income countries. Overall, the studies contributed 28 114 women with ovarian cancer (cases) and 94 942 women without ovarian cancer (controls), with 10 362 (37%) cases from Europe and 12 817 (46%) from North America. 1423 (5%) women were younger than 35 years at diagnosis, 2696 (10%) were aged 35–44 years, 6467 (23%) were 45–54 years, 9206 (33%) were 55–64 years, and 8322 (30%) were 65 years or older. Similar percentages of cases and controls reported having ever smoked.

**Table 1:** Studies and women included

		**Number of cases/controls**	**Median year of diagnosis of cases**	**Median year of birth of cases**	**Mean age at diagnosis of cases (years)**
**19 prospective studies**					
	Oxford/FPA (UK)^12^	49/196	1988	1937	48·1
	BCDDP (USA)^37^	220/1184	1991	1925	65·3
	Nurses' Health Study (USA)^46^	663/2707	1991	1930	58·7
	RCGP (UK)^14^	176/704	1991	1936	52·8
	IOWA Women's Health (USA)^28^	175/705	1991	1924	68·1
	Radiation technologists (USA)^36^	45/177	1992	1945	47·6
	Netherlands Cohort^26^	261/1805	1992	1923	67·9
	CNBSS (Canada)^27^	483/1932	1993	1932	59·2
	Norwegian Counties^30^	130/520	1993	1937	55·1
	CPS-II Mortality (USA)^21^	2554/10 718	1994	1923	70·3
	CPS-II Nutrition (USA)^38^	349/1399	1997	1929	67·7
	Southern Swedish^25^	73/293	1997	1938	57·4
	WLH (Norway/Sweden)^43^	106/427	1998	1947	48·8
	NIH-AARP (USA)^47^	763/3052	1999	1932	65·9
	EPIC (Europe)^50^	769/3099	2000	1939	59·8
	NOWAC (Norway)^29^	78/326	2000	1937	61·4
	PLCO (USA)^49^	202/807	2001	1933	68·2
	Swedish mammography^48^	89/498	2001	1936	65·7
	Million Women Study (UK)^41^	3608/14 341	2002	1941	61·0
	**All prospective studies**	**10 793/44 890**	**1999**	**1934**	**63·5**
**21 case–control studies with population controls**					
	Weiss (USA)^6^	298/1137	1977	1921	55·1
	Cramer I (USA)^3^	248/238	1979	1926	51·5
	CASH (USA)^16^	575/4233	1981	1937	41·9
	Whittemore (USA)^4^	234/683	1984	1933	50·5
	Shu/Brinton (China)^7^	228/229	1985	1933	48·4
	Western New York (USA)^24^	117/686	1988	1930	58·3
	Risch (Canada)^11^	450/564	1991	1934	56·7
	Green/Purdie (Australia)^18^	793/854	1992	1935	55·2
	Mosgaard (Denmark)^13^	907/1071	1992	1943	45·9
	Cramer II (USA)^15^	563/525	1993	1942	51·1
	Riman (Sweden)^32^	802/3361	1994	1932	61·6
	German OCS^22^	281/533	1995	1937	55·1
	Pike/Wu (USA)^31^	477/660	1995	1939	55·5
	Goodman/Wu (USA)^23^	720/895	1996	1942	55·0
	NISOC study (Israel)^19^	1342/2262	1996	1938	56·6
	OVCARE (USA)^40^	378/1637	1996	1950	45·7
	SHARE (USA)^20^	767/1367	1996	1943	51·6
	Newcomb (Two States; USA)^39^	498/3163	1998	1942	55·0
	Polish Study^42^	299/1994	2002	1947	55·4
	AOCS (Australia)^44^	1426/1492	2004	1946	56·6
	HOPE (USA)^51^	670/1551	2005	1948	57·1
	**All with population controls**	**12 073/29 135**	**1995**	**1940**	**53·8**
**11 case–control studies with hospital controls**					
	Newhouse (UK)^2^	293/597	1973	1918	54·2
	Booth (UK)^5^	288/491	1980	1927	50·9
	Tzonou/Tricopoulos (Greece)^10^	150/249	1980	1924	55·5
	Rosenberg (USA)^34^	950/3808	1983	1935	49·5
	Negri/Franceschi (Italy)^9^	972/2481	1986	1932	53·1
	WHO (developing)^8^	177/6474	1986	1943	40·0
	PEDS (USA)^35^	418/1765	1989	1933	54·7
	Negri (Italy)^17^	1031/2408	1995	1939	54·9
	Zhejiang-Curtin (China)^33^	287/652	1999	1952	46·3
	Johannesburg (South Africa)^45^	182/1492	2001	1945	54·7
	Guangzhou (China), unpublished	500/500	2006	1947	58·6
	**All with hospital controls**	**5248/20 917**	**1990**	**1937**	**52·7**
All 51 studies		28 114/94 942	1995 (1991–2000)	1937 (1928–45)	57·3 (12·4)

Data in parentheses are IQR or SD.

**Figure 1:**
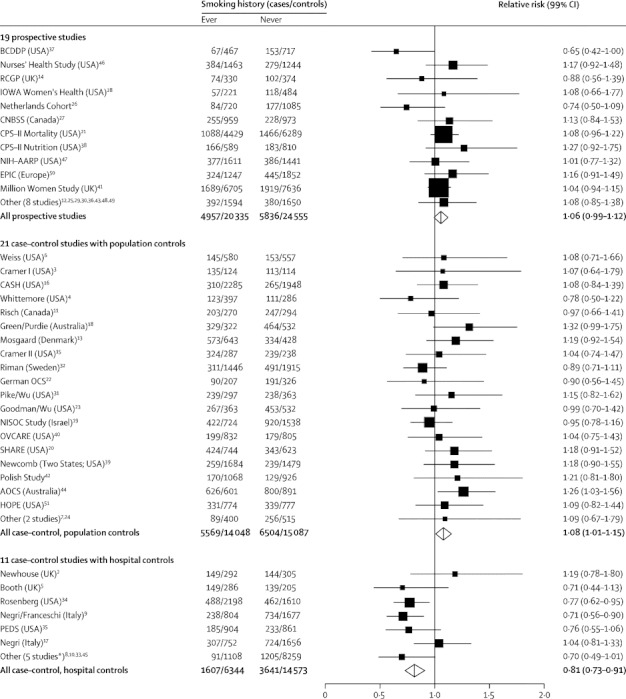
Relative risk of ovarian cancer in ever versus never smokers Stratified by study, age at diagnosis, menopausal status or hysterectomy, body-mass index, and ever use of hormonal therapy and adjusted for parity and duration of oral contraceptive use. *Including one unpublished study (Guangzhou, China).

Figure 1 shows study-specific results for ever versus never smokers, with studies grouped by design. The findings varied significantly by study design (p_heterogeneity_<0·0001) with a slightly increased risk of ovarian cancer in ever-smokers in prospective studies (RR 1·06, 95% CI 1·01–1·11, p=0·02) and in case–control studies with population controls (RR 1·08, 95% CI 1·03–1·14, p=0·003), but an apparent reduced risk in studies with hospital controls (RR 0·81, 95% CI 0·75–0·89, p<0·0001). We could not exclude the possibility that hospital controls were more likely to have conditions associated with smoking and thus not be representative of smoking habits in the general population and so we omitted studies with hospital controls from all subsequent analyses.

After excluding studies with hospital controls, we found a small increase in the risk of ovarian cancer in ever smokers compared with never smokers (RR 1·07, 95% CI 1·03–1·10, p<0·0001). There was no significant heterogeneity in the RR estimates between prospective studies and case–control studies with population controls, nor between studies within each of these designs (p>0·05 for all comparisons). All studies were of incident ovarian cancer except one (of fatal ovarian cancer^21^) and results were similar for both incident and fatal disease (figure 1).

**Figure 2:**
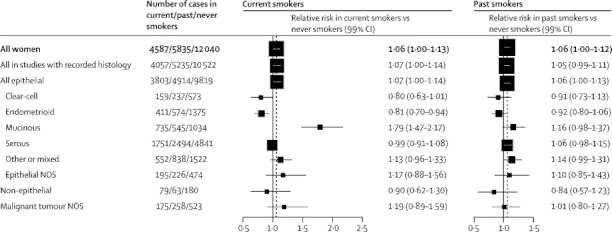
Relative risk of subtypes of ovarian cancer in current and past smokers compared with never smokers Stratified by study, age at diagnosis, menopausal status or hysterectomy, body-mass index, and ever use of hormonal therapy and adjusted for parity and duration of oral contraceptive use. Case–control studies with hospital controls were excluded. The dotted line represents the overall result for all women. NOS=not otherwise specified.

After further exclusion of the studies unable to differentiate between current and past smokers, the RR of ovarian cancer in ever smokers was 1·06 (95% CI 1·03–1·09) and was similar in current (1·06, 95% CI 1·01–1·11, p=0·01) and in past smokers (1·06, 95% CI 1·02–1·11, p=0·003) (figure 2). Of the 22 462 cases of ovarian cancer in figure 2, almost 90% (19 814) were from studies that had recorded information about tumour histology, and the RR estimates for current and past smokers were similar when analyses were restricted to these women. Most cases in studies with recorded histology were epithelial; table 2 shows information about tumour subtype and malignant potential. There was a 10-year range in mean age at diagnosis by subtype, from mean 58·6 (SD 10·4) years in women with fully malignant serous tumours to 48·8 (13·5) years in those with borderline malignant serous tumours.

**Table 2:** Distribution and characteristics of subtypes of ovarian cancer in studies with recorded histology

		**Number of cases**	**Age at diagnosis (years)**	**Year of diagnosis**
**Cases in studies with recorded histology**				
All		19 814	57·1 (11·6)	1996 (7)
Non-epithelial		322	49·3 (16·2)	1994 (8)
Epithelial		18 536	57·0 (11·5)	1996 (7)
Malignant NOS		956	61·2 (10·8)	1998 (7)
Epithelial cases*				
Clear-cell		969	56·7 (9·5)	1996 (7)
Endometrioid		2360	56·8 (10·3)	1996 (6)
Mucinous		2314	52·4 (13·1)	1995 (7)
	Fully malignant	1311	53·7 (12·9)	1995 (7)
	Borderline malignant	984	50·3 (13·1)	1996 (7)
Serous		9086	57·3 (11·5)	1996 (7)
	Fully malignant	7498	58·6 (10·4)	1996 (7)
	Borderline malignant	1389	48·8 (13·5)	1995 (7)
Other		2369	59·5 (10·3)	1997 (6)
Mixed		543	57·8 (11·0)	1999 (6)
Epithelial NOS		895	59·9 (10·9)	1996 (4)

Data are n or mean (SD). Data from case–control studies with hospital controls and studies unable to differentiate between past and current smoking were excluded. NOS=not otherwise specified.

* Fully malignant or borderline malignant status was not known for all cases; there were only two borderline malignant clear-cell, 36 borderline malignant endometrioid, and five borderline malignant mixed tumours.

In current versus never smokers, RRs varied substantially by histological subtype of the tumour (figure 2). For mucinous tumours, risk was significantly increased in current versus never smokers (RR 1·79, 95% CI 1·60–2·00, p<0·0001), but for endometrioid and for clear-cell tumours, there were significantly reduced risks (RR 0·81, 95% CI 0·72–0·92, p=0·001, and RR 0·80, 95% CI 0·65–0·97, p=0·03, respectively). Serous tumours were not significantly associated with current smoking (RR 0·99, 95% CI 0·93–1·06, p=0·8). The differences in smoking-related risk across these four specific subtypes of epithelial ovarian cancer were significant (heterogeneity p<0·0001).

**Figure 3:**
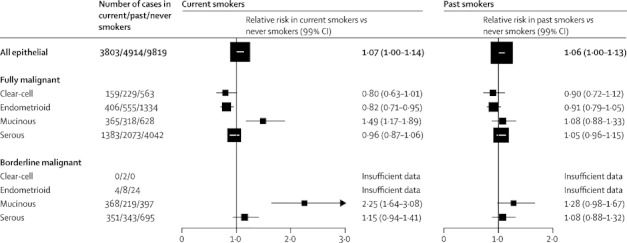
Relative risk of clear-cell, endometrioid, mucinous, and serous epithelial ovarian tumours by malignant potential and smoking history Stratified by study, age at diagnosis, menopausal status or hysterectomy, body-mass index, and ever use of hormonal therapy and adjusted for parity and duration of oral contraceptive use. Case–control studies with hospital controls were excluded. The numbers do not always match those in figure 2 because of a few cases with missing information about malignant potential.

**Figure 4:**
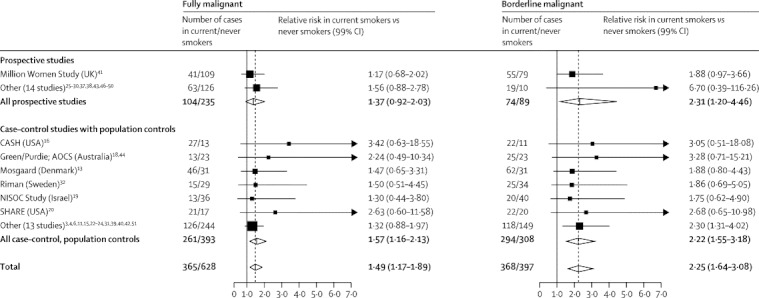
Relative risk of mucinous ovarian cancer in current versus never smokers by study Stratified by study, age at diagnosis, menopausal status or hysterectomy, body-mass index, and ever use of hormonal therapy and adjusted for parity and duration of oral contraceptive use. Case–control studies with hospital controls were excluded. The dotted line represents the overall result for all women.

The association between mucinous ovarian cancer and current smoking varied further when cancers were subdivided by their malignant potential (figure 3). The increased risk was much greater for borderline malignant (RR 2·25, 95% CI 1·91–2·65) than for fully malignant mucinous cancers (RR 1·49, 95% CI 1·28–1·73) (heterogeneity p=0·01). Neither the increased risk of borderline malignant nor that of fully malignant mucinous tumours in current smokers were driven by the findings in any one study or groups of studies (figure 4). For serous tumours, the difference in risk between borderline malignant and fully malignant cancers in current smokers was not significant (RR 1·15, 95% CI 0·99–1·33, and RR 0·96, 95% CI 0·89–1·04; heterogeneity p=0·4; figure 3). Only 36 borderline malignant endometrioid tumours were reported, which was too few to study reliably.

For past smokers compared with never smokers, there was a significant increase in the risk of borderline malignant mucinous cancers (RR 1·28, 95% CI 1·06–1·53, p=0·009), but not in the risk of fully malignant mucinous tumours (RR 1·08, 95% CI 0·93–1·26, p=0·3). There was no material increase or decrease in risk of other ovarian cancer subtypes in past versus never smokers (figure 3).

All analyses in Figure 2, Figure 3, Figure 4 were stratified by age, study, use of menopausal hormone therapy, menopausal status or hysterectomy, and BMI and adjusted by parity and duration of oral contraceptive use. Additional adjustment by year of birth, ethnic origin, education, family history of ovarian or breast cancer, age at menarche, and alcohol use changed the RR estimates by less than 2%. Furthermore, the observed associations between current smoking and overall risk of ovarian cancer, and the risk in the endometrioid, mucinous, and serous subtypes, did not vary substantially by year of birth, age at diagnosis, ethnic origin, education, alcohol use, BMI, parity, age at menarche, use of oral contraceptives, having a first-degree relative with ovarian or breast cancer, menopausal status, hysterectomy, or use of menopausal hormone therapy (table 3). There were too few clear-cell tumours to compare reliably the association with smoking between subgroups.

**Table 3:** Relative risk of all and subtypes of ovarian cancer in current versus never smokers in various subgroups of women

		**All**		**Endometrioid**		**Mucinous**		**Serous**	
		Cases in current/never smokers*	Relative risk (99% CI)	Cases in current/never smokers*	Relative risk (99% CI)	Cases in current/never smokers*	Relative risk (99% CI)	Cases in current/never smokers*	Relative risk (99% CI)
All women		4587/12 040	1·06 (1·00–1·13)	411/1375	0·81 (0·70–0·94)	735/1034	1·79 (1·47–2·17)	1751/4841	0·99 (0·91–1·08)
Year of birth									
	Before 1935	1533/5419	0·99 (0·90–1·09)	130/523	0·87 (0·66–1·14)	160/306	1·92 (1·27–2·91)	482/1949	0·86 (0·74–1·00)
	1935 or later	3054/6621	1·11 (1·03–1·20)	281/852	0·79 (0·66–0·95)	575/728	1·75 (1·40–2·18)	1269/2892	1·05 (0·94–1·17)
Age at diagnosis									
	<60 years	2925/5568	1·09 (1·00–1·18)	296/793	0·78 (0·66–0·93)	569/644	1·77 (1·41–2·23)	1226/2404	1·06 (0·94–1·19)
	≥60 years	1662/6472	1·01 (0·92–1·10)	115/582	0·86 (0·66–1·13)	166/390	1·82 (1·26–2·62)	525/2437	0·88 (0·76–1·01)
Ethnic origin									
	White	3239/7784	1·08 (1·00–1·16)	304/927	0·84 (0·70–1·01)	529/623	1·89 (1·47–2·42)	1238/3087	1·04 (0·93–1·17)
	Other	214/691	1·28 (0·89–1·85)	8/80	0·48 (0·20–1·15)	24/75	1·11 (0·42–2·95)	87/258	1·26 (0·71–2·23)
Years of education									
	<13 years	2902/6664	1·06 (0·98–1·15)	202/569	0·84 (0·67–1·06)	424/510	1·65 (1·27–2·15)	1024/2324	1·00 (0·88–1·13)
	≥13 years	1249/4175	1·05 (0·93–1·18)	128/515	0·89 (0·67–1·19)	224/398	1·97 (1·36–2·85)	550/1960	0·96 (0·82–1·12)
Alcohol use									
	Any	2088/4950	1·10 (1·00–1·20)	184/548	0·85 (0·67–1·08)	310/448	1·79 (1·33–2·41)	788/2104	1·02 (0·89–1·17)
	None	919/3758	1·04 (0·91–1·19)	81/508	0·71 (0·52–0·97)	138/322	1·65 (1·08–2·51)	369/1548	0·99 (0·81–1·21)
Body-mass index									
	<25 kg/m^2^	2553/5590	1·06 (0·98–1·15)	224/604	0·83 (0·68–1·02)	424/490	1·88 (1·43–2·47)	971/2281	1·00 (0·89–1·13)
	≥25 kg/m^2^	1815/5862	1·04 (0·95–1·14)	171/718	0·78 (0·63–0·97)	272/488	1·64 (1·23–2·19)	683/2310	0·95 (0·83–1·08)
Parity									
	Parous	3534/9176	1·06 (0·99–1·13)	304/1013	0·78 (0·66–0·92)	582/751	1·87 (1·49–2·35)	1361/3769	0·99 (0·89–1·10)
	Nulliparous	922/2374	1·07 (0·90–1·27)	106/326	0·87 (0·58–1·30)	148/261	1·57 (1·00–2·47)	346/888	1·03 (0·80–1·32)
Age at menarche									
	<13 years	1767/4507	1·09 (0·98–1·21)	163/509	0·84 (0·65–1·08)	246/349	1·81 (1·27–2·57)	656/1797	0·98 (0·84–1·14)
	≥13 years	2612/7037	1·08 (1·00–1·17)	230/808	0·85 (0·69–1·04)	450/629	1·81 (1·40–2·35)	1008/2843	1·01 (0·90–1·14)
Oral contraceptives									
	Ever-use	2075/4163	1·08 (0·98–1·19)	177/512	0·73 (0·59–0·91)	427/463	1·87 (1·41–2·47)	841/1806	1·05 (0·91–1·21)
	Never-use	2202/7150	1·03 (0·94–1·12)	207/792	0·86 (0·69–1·08)	253/505	1·72 (1·25–2·36)	777/2686	0·95 (0·83–1·08)
Mother or sister with a history of ovarian or breast cancer									
	Yes	434/1356	0·98 (0·78–1·23)	51/180	0·79 (0·47–1·33)	56/97	1·51 (0·62–3·66)	196/620	0·99 (0·71–1·39)
	No	2178/6114	1·09 (0·99–1·19)	207/811	0·75 (0·61–0·91)	393/575	1·96 (1·48–2·59)	914/2688	1·02 (0·90–1·16)
Menopausal status†									
	Premenopausal	1333/2623	1·18 (1·04–1·33)	114/364	0·69 (0·54–0·89)	300/380	1·67 (1·25–2·24)	546/1104	1·14 (0·96–1·35)
	Postmenopausal	1590/4463	1·02 (0·93–1·12)	137/507	0·75 (0·59–0·95)	249/366	1·82 (1·32–2·50)	593/1693	0·99 (0·86–1·14)
Hysterectomy									
	Yes	602/1901	1·06 (0·89–1·26)	35/181	0·68 (0·42–1·09)	87/133	1·87 (1·01–3·45)	235/771	0·97 (0·74–1·27)
	No	3727/9652	1·06 (0·99–1·13)	345/1123	0·80 (0·69–0·93)	605/848	1·75 (1·43–2·14)	1406/3846	1·01 (0·92–1·11)
Menopausal hormone therapy‡									
	Ever-use	888/2733	0·95 (0·83–1·08)	98/304	0·91 (0·64–1·29)	103/164	1·73 (1·04–2·88)	348/1236	0·83 (0·69–1·00)
	Never-use	1590/4463	1·03 (0·93–1·14)	137/507	0·80 (0·62–1·03)	249/366	1·91 (1·35–2·71)	593/1693	0·97 (0·83–1·13)

In tests for heterogeneity between subgroups no p value implies <0·01. Relative risk (RR) estimates were stratified by study and age at diagnosis, and, where appropriate, menopausal status or hysterectomy, body-mass index, and ever use of menopausal hormone therapy, and adjusted by parity and duration of oral contraceptive use. Data from case–control studies with hospital controls were excluded.

* Numbers of current or never smokers do not always add to the total because of some missing values.

† Restricted to never users of menopausal hormone therapy.

‡ Analyses relating to use of menopausal hormone therapy were restricted to postmenopausal women.

There was no significant heterogeneity between prospective studies and case–control studies with population controls in the association between current smoking and ovarian cancer risk overall (heterogeneity p=0·2) or when analyses were restricted to mucinous, endometrioid, and serous tumour subtypes (heterogeneity: mucinous, p=0·2; endometrioid, p=0·4; serous, p=0·07).

## Discussion

This collaboration has brought together and reanalysed individual participant data for about 28 000 women with ovarian cancer from 51 studies of the effect of smoking on ovarian cancer incidence. These studies provide almost all the available epidemiological evidence worldwide on the topic. Although current smoking was associated with an excess of mucinous ovarian cancer, as had been reported previously,^1^ we found that the increase was mainly in tumours of borderline malignancy rather than in fully malignant tumours. Furthermore, current smoking was associated with deficits in two other subtypes of ovarian cancer—endometrioid and clear-cell tumours. Hence, smoking had little net effect on the overall ovarian cancer incidence. The significant adverse and favourable effects of current smoking were attenuated in past smokers, so past smoking had little net effect on ovarian cancer incidence.

Results of case–control studies that used hospital controls differed qualitatively from those of studies that used other designs. These differences are unlikely to be due merely to selectively inaccurate retrospective reporting of smoking, since the results differ substantially between the retrospective studies using hospital controls and the retrospective studies using population controls. Since smoking is associated with various diseases that could lead to hospital admission it is plausible that, on average, the hospital controls were more likely to smoke than were women in the general population. This difference would dilute, and could even reverse, an association between smoking and ovarian cancer risk, as suggested by figure 1. For this reason, we omitted studies with hospital controls from the main analyses. Nevertheless, to ensure that all the epidemiological information is published, details of those studies are included in table 1 and in figure 1.

Even in case–control studies with population controls there might have been some differential participation by smoking history and the retrospective reporting of smoking might have been differentially affected by the cases' knowledge that they had ovarian cancer. Although these possibilities cannot be excluded, the similarity of the findings in case–control studies with population controls and in studies with prospective recording of smoking suggests that they might not be a serious issue here.

An advantage of seeking to review all epidemiological studies of ovarian cancer with information on smoking, published and unpublished, is that this helps to avoid unduly selective emphasis on published results or on just some studies. Only a third of eligible studies have published on the association between smoking and risk of ovarian cancer, so reviews based solely on published studies could have been susceptible to publication bias. Eligible studies that did not contribute data to this collaboration, but had published on ovarian cancer risk associated with smoking,^53^, ^54^, ^56^ together contain fewer than a tenth as many cases as are included in the present analyses. Failure to include these studies would not have substantially changed the associations reported here, because their published findings are broadly similar to our findings. Despite extensive efforts to identify all studies with unpublished results, a guarantee that others do not exist is clearly impossible. Furthermore, to have completely up-to-date information from continuing prospective studies that are accumulating data beyond the time when information was contributed to this collaboration is not possible. Ongoing prospective studies will continue to accrue women with ovarian cancer, but there is no good reason to expect that these additional data will materially change the evidence that is already available.

A further advantage of bringing together worldwide evidence on the association between ovarian cancer and smoking is that large numbers of cases are needed to assess reliably whether the association varies by tumour subtype. The histological classifications used were those provided by investigators for each study. The classification of ovarian cancers by histology and by malignant potential might have varied between studies and possibly also over time. Misclassification of tumour subtype would tend to dilute RR estimates, and blur differences between them, yet sharp differences in the smoking-related risks were found (similar differences by tumour subtype were not found for other factors such as oral contraceptive use and adiposity^57^, ^59^).

The findings for different tumour subtypes were not driven by the results from any one study or group of studies and are unlikely to be due to confounding. All analyses were routinely stratified by age, study, use of menopausal hormone therapy, menopausal status, and BMI and were adjusted by parity and oral contraceptive use; additional adjustment for six other factors hardly changed the RR estimates.

The large proportional increase in risk of mucinous ovarian tumours associated with current smoking, and the differences between the proportional increases in fully malignant and in borderline malignant mucinous tumours, are both definite findings and could reflect a real effect of smoking. Moreover, the excess risk of mucinous tumours was far greater in current than in past smokers. Although borderline malignant mucinous tumours are less aggressive than fully malignant mucinous tumours, they can have microinvasive or invasive components.^60^ The proportional reductions in smoking-related risks of clear-cell and endometrioid tumours, although not as great as the proportional increase in mucinous tumours, could also be a real effect of smoking. Since information about the amount smoked and the timing of exposure was not sought systematically for this collaboration, little could be done to examine these associations further.

Smoking is known to affect the ovaries, in that smokers have an earlier menopause than do non-smokers,^61^ but this effect does not necessarily imply that smoking would affect ovarian cancer incidence or have different effects on different tumour subtypes. The findings here support the view that different subtypes of ovarian cancer might originate in different types of cells. In particular, endometrial cancer risk is reduced in smokers^62^ and our finding of a reduced risk of endometrioid tumours in current smokers is consistent with the hypothesis that endometrioid ovarian cancers might have their origin in endometrial cells. No equivalent analogy exists for clear-cell tumours.

Smoking has a wide range of adverse effects resulting in large increases in mortality from many specific causes.^63^ Although the excess of mucinous tumours in smokers is definite, it seems to be counterbalanced by a small deficit in clear-cell and endometrioid tumours. Hence, the overall increase in incidence of ovarian cancer in smokers is small and, even in this extensive dataset, barely significant. This study could not address survival, but since about half the mucinous tumours in smokers were of borderline malignancy, smoking is likely to have little net effect on mortality from ovarian cancer.
